# Damage Identification in Various Types of Composite Plates Using Guided Waves Excited by a Piezoelectric Transducer and Measured by a Laser Vibrometer

**DOI:** 10.3390/s19091958

**Published:** 2019-04-26

**Authors:** Maciej Radzieński, Paweł Kudela, Alessandro Marzani, Luca De Marchi, Wiesław Ostachowicz

**Affiliations:** 1 Institute of Fluid-Flow Machinery, Polish Academy of Sciences, 80-231 Gdańsk, Poland; maciej.radzienski@imp.gda.pl (M.R.); wieslaw.ostachowicz@imp.gda.pl (W.O.); 2 Department of Civil, Chemical, Environmental and Materials Engineering, University of Bologna, 40136 Bologna, Italy; alessandro.marzani@unibo.it; 3 Department of Electrical, Electronic, and Information Engineering, University of Bologna, 40136 Bologna, Italy; l.demarchi@unibo.it

**Keywords:** laser vibrometry, SLDV, guided waves, damage detection, NDT, full wavefield processing

## Abstract

Composite materials are widely used in the industry, and the interest of this material is growing rapidly, due to its light weight, strength and various other desired mechanical properties. However, composite materials are prone to production defects and other defects originated during exploitation, which may jeopardize the safety of such a structure. Thus, non-destructive evaluation methods that are material-independent and suitable for a wide range of defects identification are needed. In this paper, a technique for damage characterization in composite plates is proposed. In the presented non-destructive testing method, guided waves are excited by a piezoelectric transducer, attached to tested specimens, and measured by a scanning laser Doppler vibrometer in a dense grid of points. By means of signal processing, irregularities in wavefield images caused by any material defects are extracted and used for damage characterization. The effectiveness of the proposed technique is validated on four different composite panels: Carbon fiber-reinforced polymer, glass fiber-reinforced polymer, composite reinforced by randomly-oriented short glass fibers and aluminum-honeycomb core sandwich composite. Obtained results confirm its versatility and efficacy in damage characterization in various types of composite plates.

## 1. Introduction

Guided Waves (GW) have received considerable attention in recent years, as a tool for damage detection and localization in both Structural Health Monitoring (SHM) systems and Non-Destructive Testing (NDT).

In the SHM system, typically an array of piezoelectric transducers (PZTs) is used for excitation and sensing. Various configurations may be found in the literature [1,2,3,4], and can be divided into two main groups: Pulse echo [5] and pitch-catch [6]. Algorithms for damage localization often utilize group velocities [7] of propagating Lamb wave modes, dispersion curves of particular wave mode [5] or full waveform inversion [8]. Alternatively, the time reversal approach is used [9,10]. The review of GW-based SHM strategies was presented by Mitra and Gopalakrishnan [11]. 

The advantage of such a system is the ability of permanent integration and online monitoring of changes in GW propagation in structural elements. The main problem of application of the array consisting of a few PZTs for damage localization, is that the damage imaging resolution can be quite low. On the other hand, the application of a very dense array of PZTs is not feasible. This problem can be alleviated by using scanning laser Doppler vibrometry.

Scanning laser vibrometry allows for measurement of guided waves in a dense grid of points across the surface of a large specimen [12]. Such a collection of signals is often called full wavefield. However, it should be noticed, that the measurement process can be time-consuming. Therefore, such a technique can be classified as NDT instead of SHM. Nevertheless, laser and optics technology progresses very fast, so it is expected that multipoint vibrometers, fast cameras, etc. may be used in the future for full wavefield measurements.

Anomalies of GW propagating in honeycomb core sandwich plates with debonding were studied numerically, as well as by using laser vibrometer data by Zhao et al. [13]. Various techniques of damage localization were developed in recent years using various phenomena like wave reflection detection [14], local standing wave [15], a local change in wavelength [16], energy distribution variations [17,18], local magnitude decrease [19], etc. GW measurement techniques, along with full wavefield processing techniques for damage detection, were summarized in the paper [20]. Usually, single PZT is used for GW excitation, but also high power laser can be used for pulsed excitation of thermo-elastic waves, which in conjunction with the laser vibrometer, leads to a complete non-contact monitoring system [21]. 

The aim of this research is the development of a versatile and robust technique for mapping anomalies in guided waves in plate-like structures, regardless of material properties. The proposed method can be used for various hidden defects visualization without any prior knowledge about tested material properties or its reference state (measurement data for the undamaged case). It gives the greatest flexibility of application among full wavefield techniques available in the literature.

The paper is organized as follows: The steps of the proposed method for hidden defects visualization are given in Section 2, followed by experimental results carried out on specimens made out of carbon fiber reinforced polymer (CFRP), glass fiber reinforced polymer (GFRP), randomly oriented short fiber reinforced polymer (SFRP) and honeycomb sandwich panel (HCSP). The results are briefly discussed in Section 4.

## 2. Method for Detection of Anomalies in Guided Wave Propagation

**Figure 1:**
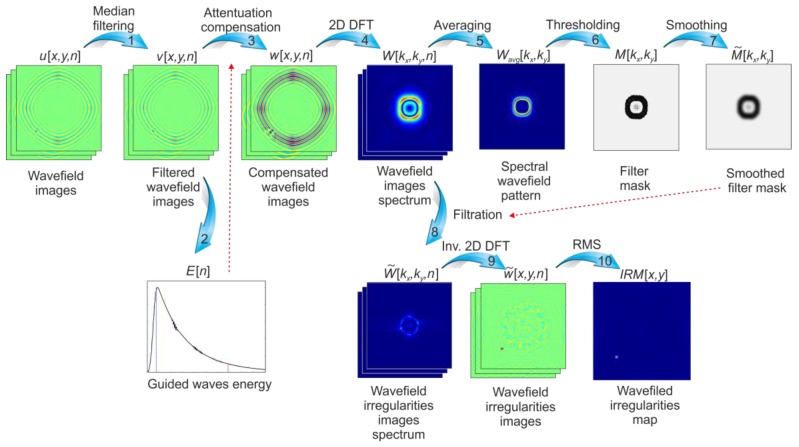
Wavefield irregularities mapping algorithm.

The method proposed in this research approach is based on the filtering technique in wavenumber domain originally proposed in [22] for identification of cracks in the plate-like structure, which was later modified for impact-induced damage detection by Kudela et al. [23]. The method is further expanded in this paper for the identification of any anomalies in propagating GW in a broad range of isotropic and anisotropic structures. The modification allows for a greater range of applicability of the proposed damage identification algorithm. Mapping irregularities in propagating GW may be used as a tool for any material local changes characterization (e.g., fatigue crack, delamination, debonding), without any prior knowledge about material properties or its reference state. It is a multi-step process. Each step of the algorithm is schematically presented in Figure 1. All the steps are indicated by arrows with consecutive numbers, and the corresponding description of each step is presented in the next sections.

### 2.1. Median Filtering

Full wavefield signals obtained by scanning laser vibrometers (out-of-plane velocity) tends to have a small number of points which have a much lower signal-to-noise ratio (SNR), so-called ‘speckle noise’. The reason for that is the fact that at some measurement points, laser light is reflected and diffused in such a way that almost no light is returning back to the scanning head. To remove this type of noise, two-dimensional median filtering is applied to every wavefield image *s*[*x,y,n*] at time sample *n* as:

(1) $$v\left[x,y,n\right]=med_{k}\left\{s\left[x,y,n\right]\right\},$$

where *med_k_*{} stands for a spatial (*x*, *y*) median filtering operation with moving *k* × *k* size window. Presented in Section 3, results were obtained using 2 × 2 size median filter. It is worth noticing that large median filter window sizes (e.g., k > 4) should be avoided, so as not to distort the propagating GW profile, which may lead to false damage identification. 

### 2.2. Energy Function 

In this step, the total signal energy *E*[*n*] of the wavefield images for every time sample *n* is determined as:

(2) $$E\left[n\right]=\overset{N}{\underset{x=1}{\sum }}\overset{M}{\underset{y=1}{\sum }}v{\left[x,y,n\right]}^{2},$$

where *N* and *M* are the numbers of points in *x* and *y* directions, respectively. 

### 2.3. Attenuation Compensation

As the guided waves propagate through the tested material, their total energy is decreasing due to dissipation (material friction) and energy transfer to surroundings. This phenomenon causes difficulties in the identification of damage located further form the excitation point as well as ambiguity in damage severity estimation. In order to minimize this effect, a normalization function given as *E_r_*[*n*] is introduced:

(3) $$E_{r}\left[n\right]=\{\begin{array}{ll} \frac{E\left[n\right]}{E_{max}} & \mathrm{for}\;n\geq n_{a}\\ 1 & \mathrm{otherwise} \end{array}.$$

where *E_max_* = max(*E*[*n*]). The normalization is applied to the full wavefield, so that guided full wavefield signal with compensated attenuation *w*[*x,y,n*], is obtained

(4) $$w\left[x,y,n\right]=\frac{v\left[x,y,n\right]}{E_{r}\left[n\right]}.$$

**Figure 2:**
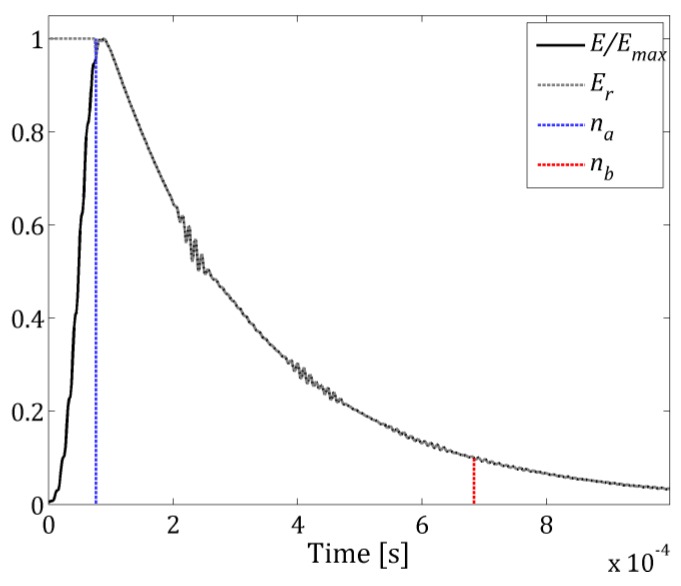
Normalized Guided Waves (GW) energy function *E/E_max_* and attenuation compensation function *E_r_*.

The exemplary normalization function *E_r_* is presented in Figure 2 (grey dashed line).

Normalized to unity energy function *E*/*E_max_* (Figure 2—black line) is also used to define the time interval between time sample *n_a_* (Figure 2—blue dashed line), when the signal has maximum energy, and *n_b_* (Figure 2—red dashed line), when its energy drops to a given threshold (e.g., 10%). This interval is regarded as most advantageous for wavefield irregularities mapping, and it is used further in Equation 16.

### 2.4. Two-Dimensional Discrete Fourier Transform

All wavefield images are transformed from the space-space-time domain into a wavenumber-wavenumber-time domain by two-dimensional discrete Fourier transform (2D DFT)

(5) $$W\left[k_{x},k_{y},n\right]=\frac{1}{\sqrt{MN}}\overset{N}{\underset{x=1}{\sum }}\overset{M}{\underset{y=1}{\sum }}v\left[x,y,n\right]\cdot e^{-i2\pi \left(\frac{k_{x}x}{N}+\frac{k_{y}y}{M}\right)},\;n\in \mathbb{N}:\langle 1,P\rangle ,$$

where *k_x_* and *k_y_* are wavenumbers in the *x* and *y* directions, respectively. 2D DFT is applied for each time frame-wavefield image, therefore *P* transformations are performed, were *P* is the number of wavefield images (number of registered time samples in each point).

### 2.5. Spectral Wavefield Pattern 

In wavenumber domain a spectral wavefield pattern is determined through averaging a set of wavefield images in the time interval between *n_c_* and *n_d_*:

(6) $$W_{avg}\left[k_{x},k_{y}\right]=\frac{1}{n_{d}-n_{c}}\overset{n_{d}}{\underset{n=n_{c}}{\sum }}W\left[k_{x},k_{y},n\right],$$

where *n_c_* and *n_d_* are determined as:

(7) $$n_{c}=n_{a}-n_{f},$$

(8) $$n_{d}=n_{b}+n_{f}.$$

Value of *n_f_* is chosen to meet the conditions:

(9) $$\frac{E\left[n_{a}-n_{f}\right]}{E_{max}}\approx 0.5\;n_{f}\in \mathbb{N}:\;n_{f}<n_{a}.$$

**Figure 3:**
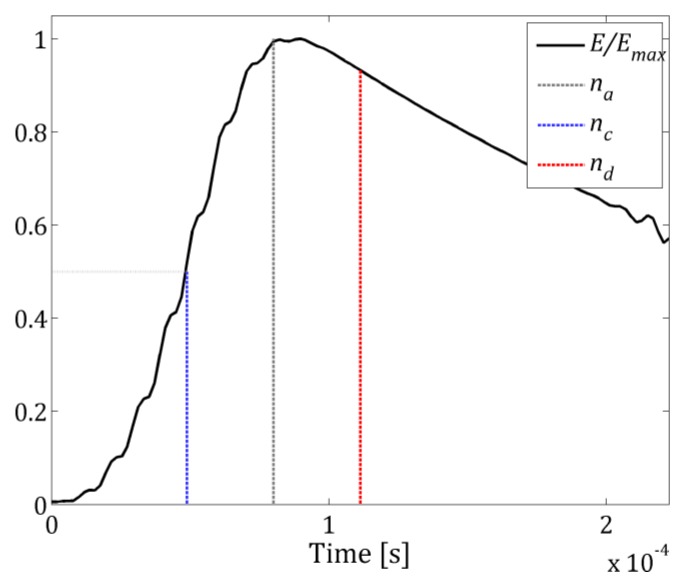
Normalized GW energy function *E/E_max_* with marked by blue and red dashed line time interval used for wavefield pattern estimation.

The exemplary *E/E_max_* function with *n_a_*, *n_c_* and *n_d_* position marked are presented in Figure 3.

### 2.6. Filter Mask

Based on the spectral wavefield pattern a filter mask *M* is determined as:

(10) $$M\left[k_{x},k_{y}\right]=\{\begin{array}{cc} 0\; & if\;W_{avg}\left(k_{x},k_{y}\right)>\mathrm{threshold}\\ 1 & \mathrm{otherwise} \end{array}.$$

The threshold is chosen to be the 95th percentile of *W_avg_* values. It may be determined by finding the *W_avg_* value for which its cumulative distribution function reaches 0.95. If the threshold will be too high (not enough points will be filtered), some portion of the regular wave propagation component will not be filtered, thus making final irregularities map to have high values across the whole inspected region. On the other hand, setting threshold too low (too many points will be filtered) will result in blurred irregularities mapping up to the point that they are removed from the signal completely. However, even a wide range of threshold values around the 95th percentile of *W_avg_* values will give good results. 

### 2.7. Smoothing Filter Mask

A rotationally symmetric Gaussian low pass filter *G* is used to smooth filter mask *M* throughout the convolution operation

(11) $$\tilde{M}\left[k_{x},k_{y}\right]=M\left[k_{x},k_{y}\right]*G\left[k_{x},k_{y}\right].$$

The standard deviation *σ* and size of the filter may be chosen from a wide range of values. In this study *σ* = 2 and 10 × 10 points size *G* were used.

(12) $$G\left[k_{x},k_{y}\right]=\frac{g\left[k_{x},k_{y}\right]}{\sum_{k_{x}}\sum_{k_{y}}g\left[k_{x},k_{y}\right]}\;,$$

where

(13) $$g\left[k_{x},k_{y}\right]=e^{\frac{-\left(k_{x}{}^{2}+k_{y}{}^{2}\right)}{2\sigma^{2}}}.$$

### 2.8. Filtering 

A designated smoothed filter mask $\tilde{M}$ is successively used for the elimination of main wavefield components in every wavefield image in the wavenumber domain by element-wise multiplication as:

(14) $$\tilde{W}\left[k_{x},k_{y},n\right]=W\left[k_{x},k_{y},n\right]\circ \tilde{M}\left[k_{x},k_{y}\right],\;\;n\in \mathbb{N}:\langle 1,P\rangle .$$

This operation removes the regular component of GW, leaving an irregular component of wavefield images caused by abnormalities in GW-like local changes in wavelength, amplitude, wavefront orientation, or the occurrence of reflection. 

### 2.9. Inverse Two-Dimensional Discrete Fourier Transform 

To transform the wavefield irregularities images from the wavenumber-wavenumber-time domain back into the space-space-time domain, an inverse two-dimensional discrete Fourier transform (Inv. 2D DFT) is applied for each wavefield image (time frame) as:

(15) $$\tilde{w}\left[x,y,n\right]=\frac{1}{\sqrt{MN}}\overset{N}{\underset{k_{x}=1}{\sum }}\overset{M}{\underset{k_{y}=1}{\sum }}\tilde{W}\left[k_{x},k_{y},n\right]\cdot e^{i2\pi \left(\frac{k_{x}x}{N}+\frac{k_{y}y}{M}\right)},\;n\in \mathbb{N}:\langle 1,P\rangle .$$

### 2.10. Root Mean Squared 

In step 9, a series of processed images for consecutive time moments are obtained. To fuse information about any abnormality in wavefield images into a single map, the Root Mean Squared (RMS) function is used. The final wavefield irregularities map is given as follows:

(16) $$IRM\left[x,y\right]=\sqrt{\frac{1}{n_{b}-n_{a}}\overset{\;n_{b}}{\underset{n=n_{a}}{\sum }}{\left(\tilde{w}\left[x,y,n\right]\right)}^{2}}.$$

## 3. Experimental Verification

### 3.1. Experimental Set-Up

**Figure 4:**
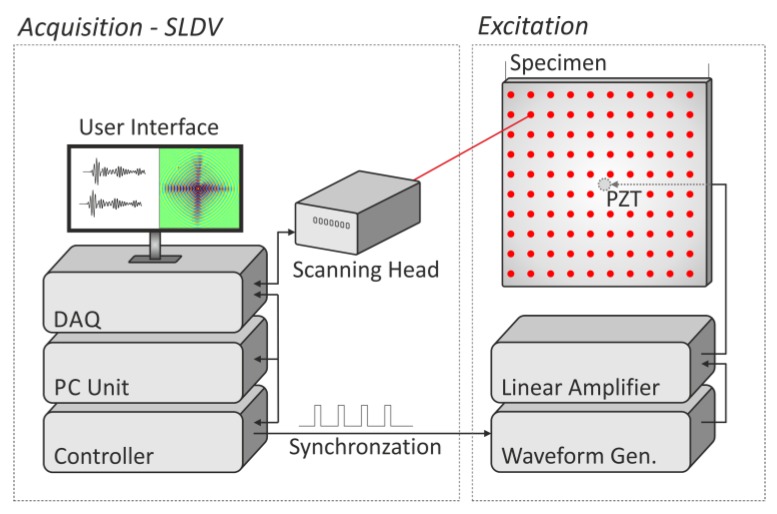
Experimental set-up.

In each tested specimen (detailed description in Section 3.2), guided waves were excited by a round piezoelectric transducer (Sonox^®^ P502 produced by CeramTec) of a 10 mm diameter. According to the producer datasheet, its thickness resonance is 3.8 MHz, and the planar resonance is 203 kHz. The transducer was attached by an acrylic glue (Kropelka^®^ made in Uruguay) to the back surface of each investigated specimen in its geometrical center. The signal in a form of 5 sine cycles with 50 kHz frequency modulated by Hann window was generated by an arbitrary waveform generator (Aim & Thurlby Thandar Instruments TGA 1241), and amplified to 200 Vpp by a Linear Amplifier (Piezosystems EPA-104). Guided waves were measured by a scanning laser Doppler vibrometer (Polytec PSV-400) as out-of-plane velocities in a regular grid of 251 × 251 points covering the whole front surface of investigated specimens. The scheme of the experimental set-up is presented in Figure 4. 

Measurements were performed in point by point manner, and were synchronized with excitation. Between each excitation 10 ms delay was used to ensure that all previously excited GW attenuated. In each measuring point, 512 time samples were registered with a 512 kHz sampling rate, which gives a total of 1 ms measured time response. Every signal was measured 10 times, and the averaged value was designed to improve signal quality. Full wavefield measurements took 6 h for each investigated specimen.

### 3.2. Specimens

**Figure 5:**
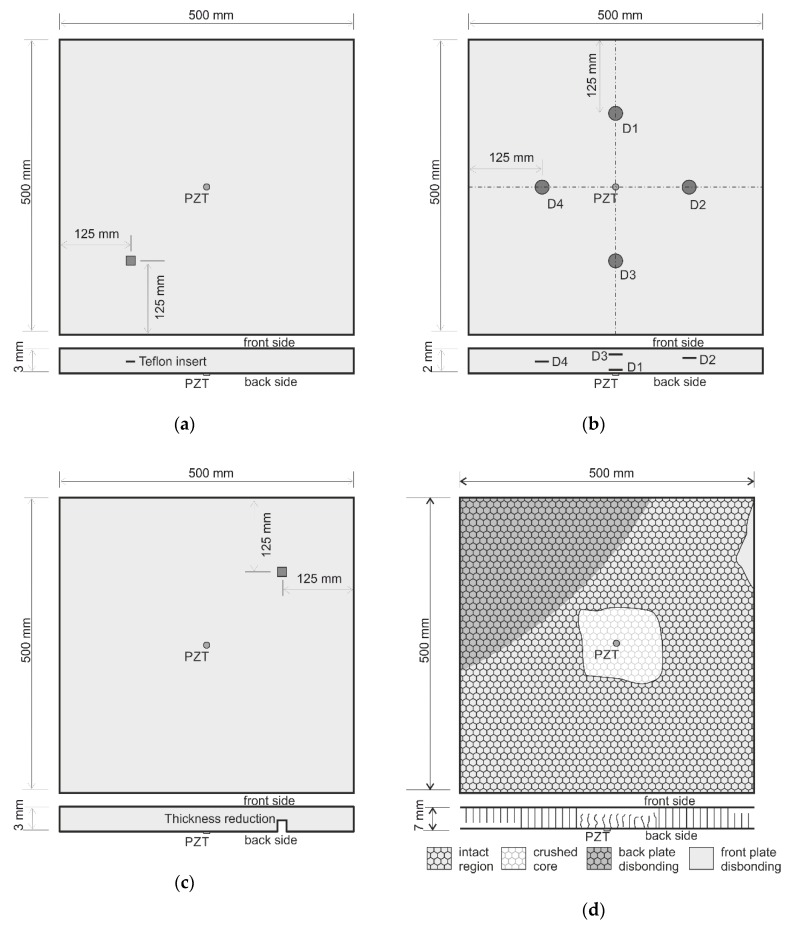
Specimens with defects schemes: (**a**) Carbon fiber reinforced polymer (CFRP); (**b**) glass fiber reinforced polymer (GFRP); (**c**) randomly oriented short fiber reinforced polymer (SFRP) and (**d**); honeycomb sandwich panel (HCSP).

In order to verify the proposed wavefield irregularities mapping technique for damage identification, four various composite plates, namely: CFRP, GFRP, SFRP, HCSP specimens with various defects were investigated. A detailed description of specimens is given in Section 3.2.1, Section 3.2.2, Section 3.2.3 and Section 3.2.4, and the schemes are presented in Figure 5.

All tested specimens front surfaces were covered with retro-reflective tape in order to increase the SNR of measured time responses. All specimens were of 500 mm width, and 500 mm length and, various thickness. Specimens were hung by a tiny string attached to its sides to reduce the external influences and simulate free-free conditions.

#### 3.2.1. CFRP

Carbon fiber reinforced polymer plate consists of 16 woven fabric layers (GG204P-IMP503 pre-pregs) with fibers orienting 0° and 90° in every layer. The total thickness of the specimen is 3 mm. A 15 × 15 mm square Teflon insert was introduced to the sample during the manufacturing process between layers 8 and 9 to simulate a delamination. The scheme of the sample with the delamination position is presented in Figure 5a.

#### 3.2.2. GFRP

Glass fiber reinforced polymer plate consists of 12 woven fabric layers (GG204P-VV192T/202 pre-pregs) with fiber orienting 0° and 90° in every layer. The total thickness of the specimen is 2 mm. Four round Teflon inserts with a diameter of 20 mm were introduced to the sample during the manufacturing process. Positions of the delaminations in thickness are given in Table 1 and presented in Figure 5b.

#### 3.2.3. SFRP

Randomly-oriented short fiber-reinforced polymer plate comprises 4 mm long glass fiber bundles (40% by weight) evenly distributed in epoxy resin (Ampreg 22). The sample has a uniform thickness of 3 mm. In this specimen, the damage was prepared as a 20 × 20 mm area of reduced thickness by 50% from the back side. The scheme of the sample with damage position is presented in Figure 5c.

#### 3.2.4. HCSP

Honeycomb sandwich panel is composed of two 1 mm thick 5005 aluminum sheets and 5 mm tall aluminum honeycomb core (3003 aluminum foil of 50 microns thickness and 12 mm cells diameter) bonded in between. In the central part of the specimen, during the manufacturing process, the aluminum core was crushed at the area of about 125 × 125 mm. Additionally, two regions where the inner core was disbonded from the outer layer were introduced. The scheme of the HCSP specimen with its deteriorated areas is presented in Figure 5d.

### 3.3. Results

#### 3.3.1. Wavefield Images

**Figure 6:**
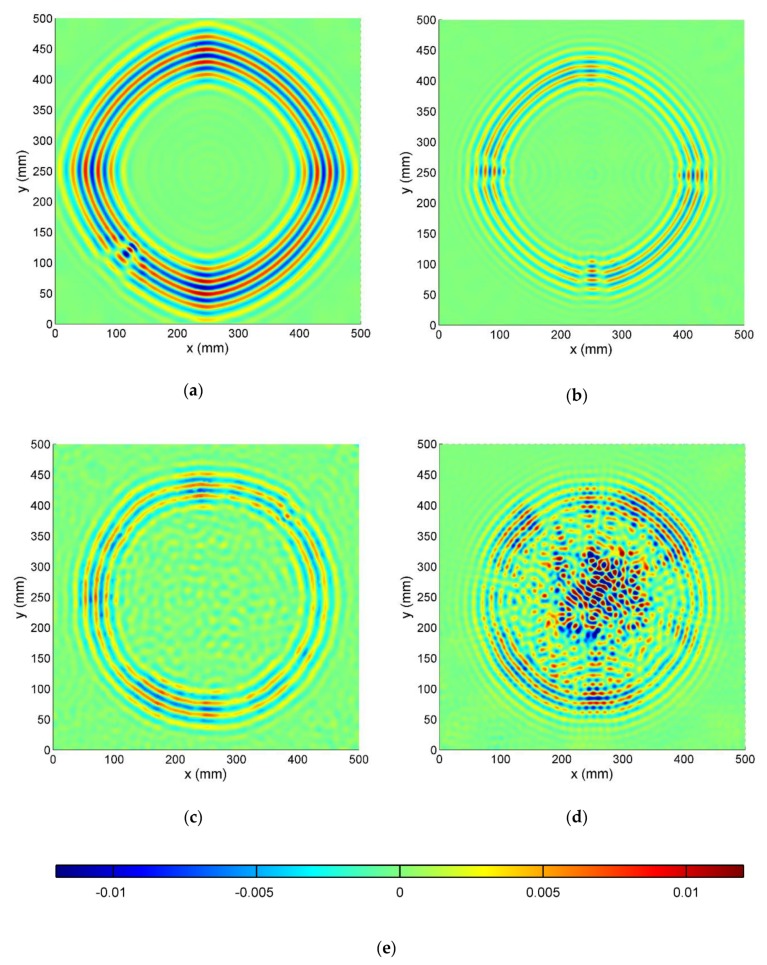
Wave images obtained at t = 200 µs for: (**a**) CFRP; (**b**) GFRP; (**c**); SFRP (**d**); HCSP specimens; (**e**) Color scale.

Median filtered wavefield images *v*[*x,y,n*] for all test specimens at one chosen time moment are presented in Figure 6. Due to relatively low excitation frequency, namely 50 kHz, only fundamental A0 and S0 modes are propagating in the investigated structures. It should be noted that in the antisymmetric A0 mode, transverse particle motion dominates whereas, in the symmetric S0 mode, in-plane particle motion dominates. Since in this study, out-of-plane velocity components of propagating waves were measured, in all presented cases A0 mode is dominating over S0 mode.

In Figure 6a (CFRP specimen) wavefront of propagating GW is smooth and elongated in the horizontal and vertical direction, which corresponds to fibers orientation. The occurrence of damage is visible in the left bottom quarter of the image as a local disturbance of wavelength and amplitude.

In Figure 6b (GFRP specimen) wavefront is almost circular with evident distortions at four quadrants, where the delaminations are located. Also reflected from Teflon inserts, small amplitude waves may be noticed inside the circular shape. The smallest changes in wavefront are visible in the top quadrant (delamination D1) where the Teflon insert is located farthest from the measured surface.

In Figure 6c (SFRP specimen) wavefront is rugged both in terms of wave position (related to wave velocity) and amplitude. Small amplitude GW reflections are spread across the whole specimen surface. Therefore, damage position (top-right quarter) is hard to be distinguished.

In Figure 6d (HCSP specimen) the circular wavefront has a strong variation in the amplitude along its circumference. High amplitude wave reflections occur in the specimen’s center part where the crushed core area is located.

**Figure 7:**
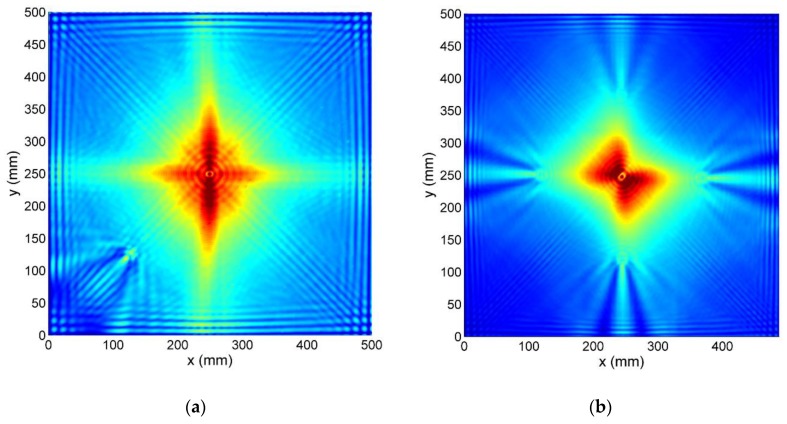

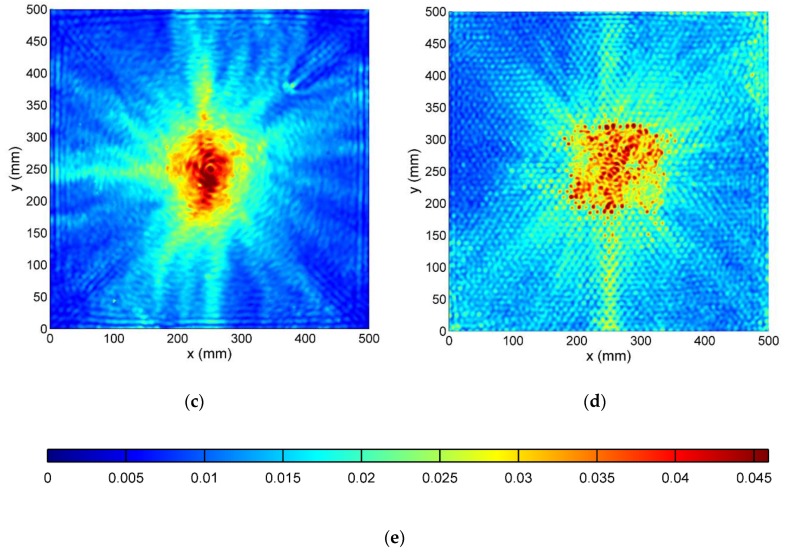
RMS maps for: (**a**) CFRP; (**b**) GFRP; (**c**); SFRP (**d**); HCSP specimen; (**e**) Color scale.

**Figure 8:**
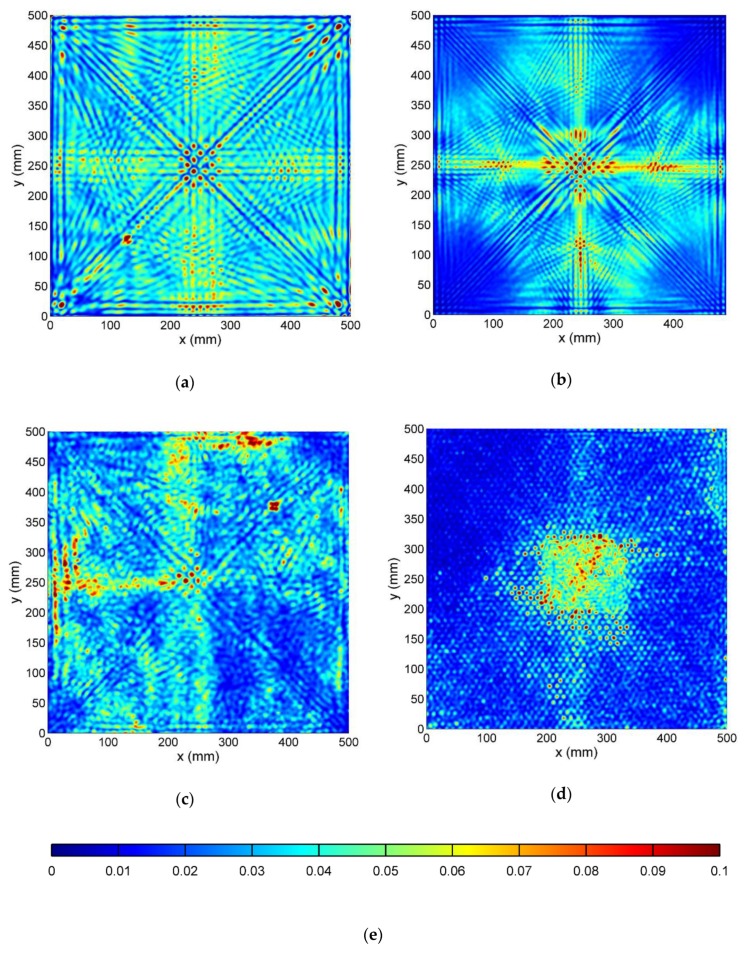
Attenuation-compensated RMS maps for: (**a**) CFRP; (**b**) GFRP; (**c**); SFRP (**d**); HCSP specimen; (**e**) Color scale.

RMS maps of tested specimens calculated for median-filtered registered full wavefield signals *v*[*x,y,n*] are presented in Figure 7. Due to high GW attenuation in tested materials, most of the energy of the wave is concentrated around the excitation point. For comparison purposes also, RMS maps determined for compensated signals *w*[*x,y,n*], are presented in Figure 8.

In the CFRP sample (Figure 7a) energy of GW is spread more in fibers directions, creating a cross-like shape in the center. This phenomenon is less apparent in GFRP specimen (Figure 7b), where besides this effect also, energy is distributed more in the direction of two corners (top-left corner and bottom-right corner). This is most probably caused by asymmetry in the PZT transducer, which has one of the electrodes wrapped around from the bottom to the top surface. 

It is worth it to notice that all Teflon inserts (CFRP and GFRP specimens) create similar patterns in RMS maps. Higher amplitude beams are formed behind delaminations in a straight line for excitation point, and some small side lobes on both its sides are separated by lower value regions.

For SFRP specimen, RMS map (Figure 7c) is much more scattered, and damage position is much less evident, but it is creating a similar pattern to the case of delaminations. 

The crushed core region of HCSP appears as a high-value area in the RMS map (Figure 7d). Front plate disbonding has a small increase in RMS amplitude, and back plate disbonding manifests itself as lower amplitude area.

#### 3.3.2. Spectral Wavefield Patterns

**Figure 9:**
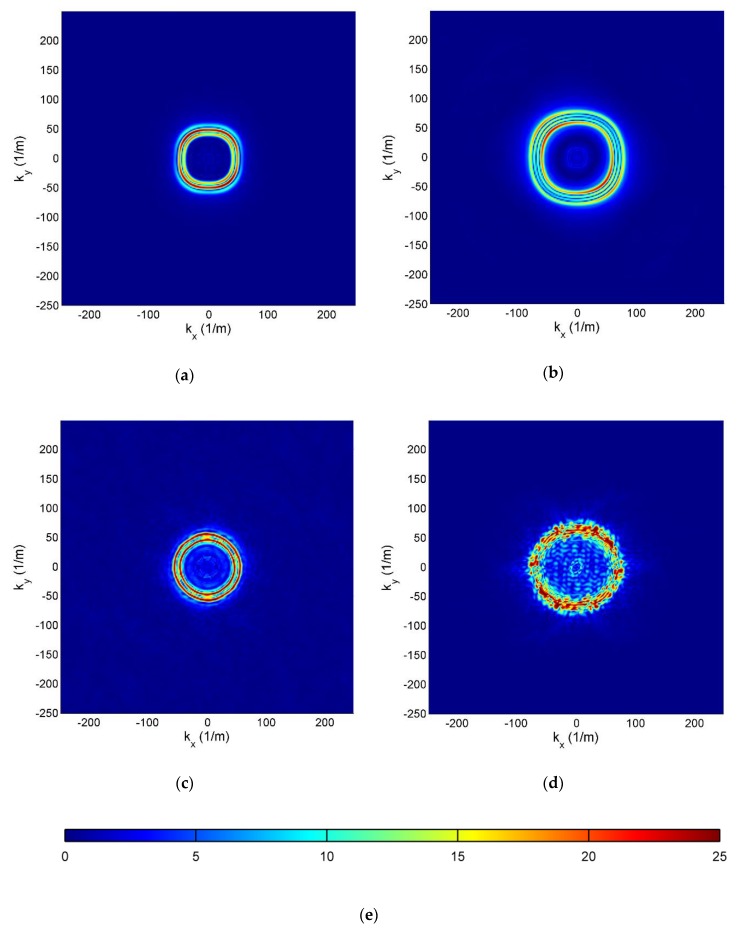
Wavefield patterns in wavenumber domain for: (**a**) CFRP; (**b**) GFRP; (**c**); SFRP (**d**); HCSP specimen; (**e**) Color scale.

Spectral wavefield patterns *W_avg_* in a form of magnitude maps are presented in Figure 9. Despite the fact that the same excitation was applied to each specimen, wave patterns are diversified, which is due to the difference in material properties and specimen thickness. In CFRP and GFRP, smooth oblate ring shapes are visible, in SFCP an almost round wavefield pattern with complex blurred background around it, and the most complex pattern occurs in HCSP.

**Figure 10:**
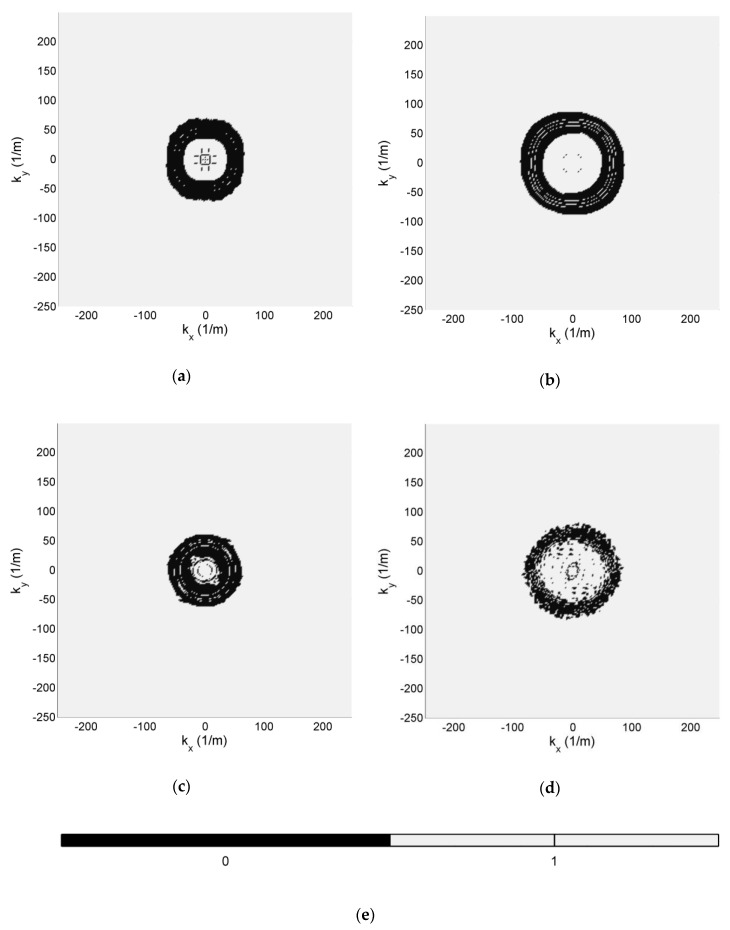
Filter masks for: (**a**) CFRP; (**b**) GFRP; (**c**); SFRP (**d**); HCSP specimen; (**e**) Color scale.

By thresholding of wavefield patterns, filter masks were determined, and are presented in Figure 10, where the black areas stand for 0 value, and they light grey areas stand for 1.

#### 3.3.3. Wavefield Irregularities Maps

**Figure 11:**
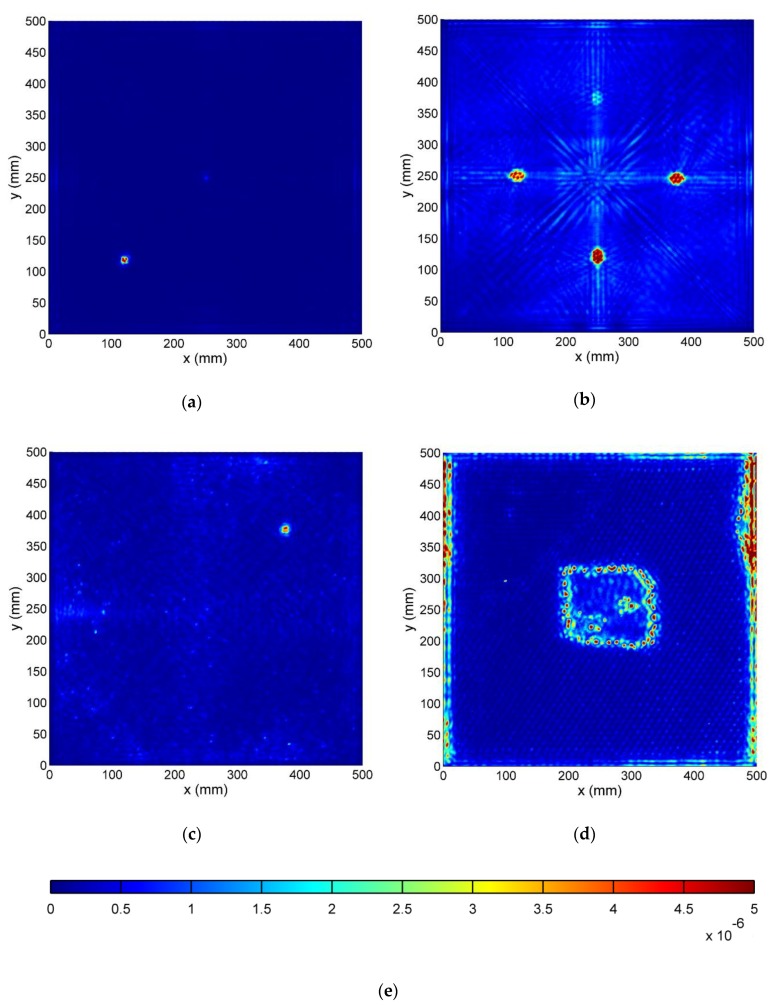
Wavefield irregularities map in: (**a**) CFRP; (**b**) GFRP; (**c**); SFRP (**d**); HCSP specimen; (**e**) Color scale.

Final maps of wavefield irregularities are presented in Figure 11. 

Teflon insert in the CFRP specimen (Figure 11a) is clearly visualized with its proper location, size, and shape. Small amplitude values of the map in the center correspond to the PZT transducer. 

In Figure 11b, all four delaminations are visible with the correct position and elongated by about 50%. Defect position in depth from the measured surface shows a correlation with obtained map values. The closer the delamination is to the front surface of the specimen, the higher the value on the presented map occurs.

In the SFRP specimen (Figure 11c), due to a distorted wavefield by randomly distributed fibers, small irregularities identification covers the whole specimen surface. However, the damage is clearly visible with its shape and size, corresponding to the sample specification.

Crushed honeycomb filler edges in Figure 11d are well defined. Front plate deboning is visible on the right side with additional high-value regions along the left and right edges. Only back panel disbonding cannot be identified from these obtained maps. Its edge is barely visible and small change in a dotted pattern inside this region may be noticed.

## 4. Discussion

In this work, a technique for mapping irregularities of propagating guided waves is proposed. It is used for damage detection, localization, and assessment. The effectiveness of this method was verified experimentally on four various composite plates with defects. Guided waves were excited by PZT transducer, and measured by scanning laser vibrometer. 

The main advantage of the proposed algorithm is its robustness, versatility and high efficacy. It is fully automatized, giving results easy to interpret. No *a priori* information about material properties or reference data is needed. It is suitable for the identification of a wide variety of manufacturing defects and damage that would have occurred during structure operation in various composite materials. The algorithm uses fast Fourier transform implementation, and therefore is not computationally intensive, while the processing of presented results took less than 5 s for each sample on a modern desktop computer.

Further studies should be focused on testing the proposed method on a more complex, real structure.

## Figures and Tables

**Table 1:** Positions of Teflon inserts in glass fiber reinforced polymer (GFRP) specimen.

Designation	Position in Thickness	Size
D1	between 10th and 9th layer	d = 20 mm
D2	between 8th and 7th layer	d = 20 mm
D3	between 6th and 5th layer	d = 20 mm
D4	between 4th and 3rd layer	d = 20 mm
